# An exceptional collision tumor: gastric calcified stromal tumor and pancreatic adenocarcinoma

**DOI:** 10.11604/pamj.2015.22.289.7574

**Published:** 2015-11-24

**Authors:** Hicham Baba, Mohamed Elfahssi, Mohamed Said Belhamidi, Abderrahman Elhjouji, Ahmed Bounaim, Abdelmounaim Ait Ali, Khalid Sair, Aziz Zentar

**Affiliations:** 1 Departement of surgery I, Mohamed V Teaching Military Hospital, Rabat 10100, Morocco

**Keywords:** Collision tumor, stromal tumor, adenocarcinoma

## Abstract

The authors report an exceptional case of collision tumor comprised of a gastric calcified stromal tumor and a pancreatic adenocarcinoma. The pancreatic tumor was detected fortuitously on the histological exam of resection specimen.

## Introduction

The collision tumor is defined as the existence of two different histologic types of carcinoma that are either contiguous or intermingled.

## Patient and observation

**Figure 1:**
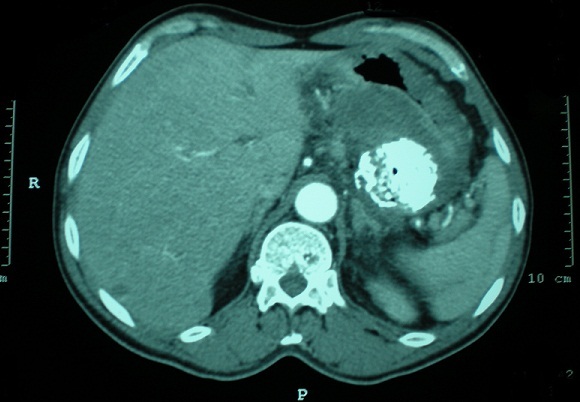
Partially calcified mass with heterogeneous contrast enhancement on the fundal region and greater curvature of the stomach

**Figure 2:**
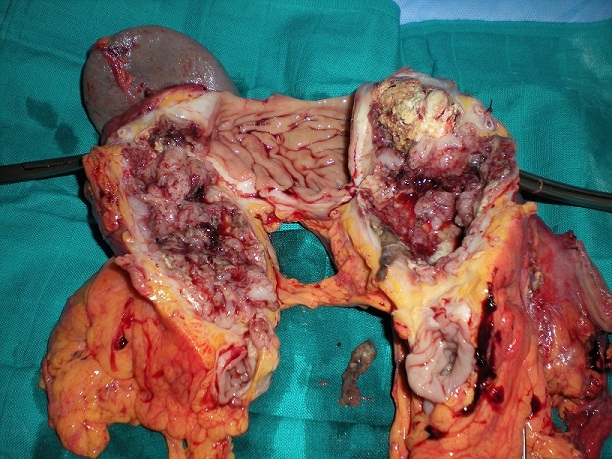
Specimen of resection

**Figure 3:**
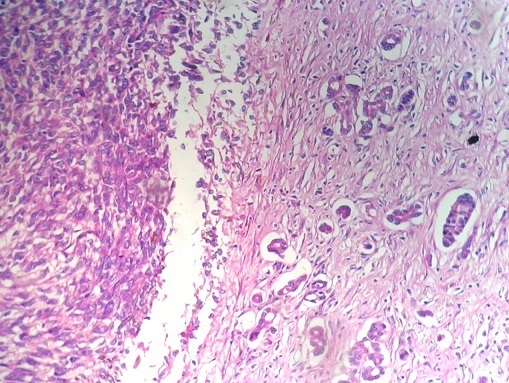
Zone of collision between the gastric stromale tumor (left) and the pancreatic adenocarcinoma (right) (HEx40)

**Figure 4:**
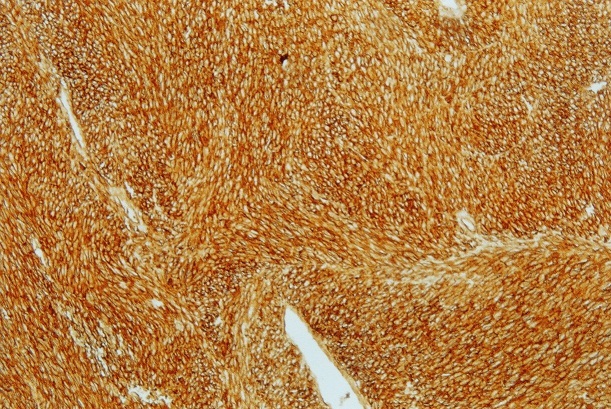
CD117 expression in stromal gastric tumor (x 10)

The patient was a 70-year-old male who presented with a 6-month history of vomiting, epigastric pain and weight loss. A gastroscopy revealed a large submucosal lesion originating in the fundus of the stomach and the biopsies were inconclusive. CT study revealed a 12x10x9 cm partially calcified mass with heterogeneous contrast enhancement on the fundal region and greater curvature of the stomach (Figure 1). The patient underwent a total gastrectomy associated to a distal splenopancreatectomy and segmental transverse colectomy (Figure 2). The final pathologic diagnosis was collision tumor of gastric GIST and pancreatic adenocarcinoma (Figure 3, Figure 4).

## Discussion

Collision tumor has been defined as two histologically differing tumors simultaneously involving the same organ with an equivocal intermediate transitional zone between them [1]. Our patient presents an exceptional case of collision tumor between two tumors arising from different organs. One case of an adenocarcinoma of the head of the pancreas associated with a low grade gastric stromal tumor was reported; however, the exact location of the gastric tumor was not specified [2]. Otherwise, the synchronous occurrence of GISTs and other primary tumors is considered an uncommon entity however such occurrence has been more frequently described in literature mainly in form of single case reports. This situation is almost always discovered incidentally as during surgery or staging exams of the primary disease. Further, GISTs have been reported to occur synchronously mostly with adenocarcinoma. Among the assumptions that have been invoked to explain synchronicity is that the environmental carcinogens might affect molecular pathways that are shared by mesenchymal and epithelial cells of the digestive tract [3]. The other particularity of our observation is GIST′s calcification which is not a usual clinicopathologic feature of this type of tumor. In a series of 29 cases, there are only three primary GISTs containing foci of calcification (10.3%) [4, 5].

## Conclusion

If collision tumor in the same organ is uncommon and usually discovered incidentally; it is exceptional when it occurs between two tumors of adjacent organs.
